# The Influence of the Penitentiary System

**Published:** 1848-07-01

**Authors:** 


### ON THE INFLUENCE OF THE PENITENTIARY SYSTEM IN INDUCING INSANITY.

M. Ferrus, at a meeting of the Academy of Science in Paris, quoted a letter of M. Bouchet, physician of the Asylum for the Insane at Nantes, relating to the influence exercised by the penitentiary system in giving rise to mental alienation. M. Collineau and M. Ferrus had previously published an abstract of the memoir of Dr. Joret, whose conclusions relate to thirty cases of mental alienation occurring in the penitentiary of Vannes, which is regulated according to the principles in force at Auburn; and of M. Bouchet's paper, which had reference to fifteen cases of insanity, which were brought under the care of that gentleman in the year 1845, from the penitentiary already mentioned.

Of these fifteen cases, one is dead, five are still in the establishment, and the remaining nine have lately been dismissed; three having been sent not cured to the Asylums for the Insane in their respective departments, on the expiration of their term of punishment. Of these three, one was imbecile and epileptic; another affected by long-existing monomania and hallucinations of hearing and sight; whilst the third was suffering from that species of reasoning or instinctive monomania which takes its origin in a lesion of the sensibility without affecting the reasoning powers sufficiently to justify the term of insanity until characterized by some acts of violence. Of the six others, three were affected by monomania which appeared recent, and was accompanied by hallucinations that might be referred to fear and terror; the three others, one of whom was primarily weak-minded, were affected by reasoning, or instinctive monomania, that had existed for a long time, and prior to their condemnation. The patient who died, wept incessantly, declaring that she had been in that state for more than ten years, although she had only been condemned two years before. Of the five still in the establishment, one affected by monomania and hallucinations of sight, had been insane three or four years before her conviction. Another had been confined as insane two years before the robbery for which she was convicted, and the remaining three were more or less affected by a sort of instinctive monomania combined with intellectual weakness. M. Bouchet here asks, whether we may conclude from these facts, that the Auburn system is an essentially productive cause of mental derangement? In his opinion, no absolute reply can be made to this question; and he thinks, with MM. Lélut and Baillarger, that mental derangement frequently precedes crimes that lead to condemnation, and even in most cases determines them, without, however, being manifested by conclusive evidence on the trial. His opinion is founded, as far as relates to the fifteen cases above cited, on the fact that there is not a single case of mania, properly so called, among them; and that, in general, the existence of madness prior to the perpetration of the offence was fully established.

With respect to the general question at issue, M. Bouchet is of opinion, that without absolutely condemning either the system of solitary confinement or the system of absolute silence, and being still less disposed to propose a new system, the following principles, which have been very generally adopted in practice, may be admitted—viz., that derangement of the mental and intellectual faculties, generally speaking, experiences a considerable degree of amelioration when the patients are allowed, under certain prescribed rules, to mix together in common, but that mental derangement of the moral faculties is aggravated by the influence of the same conditions that incessantly excite sensibility in turning it aside from its normal direction; and in these cases, M. Bouchet believes that we are justified in looking with much distrust on the system used at Auburn (that of the patients mixing together) as a corrective or curative means of treating the disease. M. Bouchet is more inclined to believe that the Pennsylvanian system is the best adapted to calm the excitement common to these cases of insanity, and to induce permanent amendment.—*Gazette Medicale,* No. XIX., 1848.

